# A 15-year experience in pediatric palliative care: a retrospective hospital-based study

**DOI:** 10.1186/s12904-024-01532-1

**Published:** 2024-08-06

**Authors:** Mirella Schiavon, Pierina Lazzarin, Caterina Agosto, Francesca Rusalen, Antuan Divisic, Anna Zanin, Anna Mercante, Valentina Mirisola, Simonetta Papa, Luca Giacomelli, Franca Benini

**Affiliations:** 1 Paediatric Palliative Care Pain Service Department of Women’s and Children’s Health, University of Padua, Padua, Italy; 2 Department of Biomedical and Neuromotor Sciences, University of Bologna, Bologna, Italy; 3 Polistudium SRL, Milan, Italy

**Keywords:** Pediatric palliative care, Follow-up, Mortality

## Abstract

**Background:**

The current Italian scenario of pediatric palliative care (PPC) services is characterized by inadequate coverage of the territory. Therefore, it is important to improve the referral of patients to the most appropriate setting (community care, general PPC, or specialized PPC) and to improve the delivery of PPC care.

**Methods:**

Aiming at obtaining information about the referrals to the Padua Pediatric Hospice that could help estimate the investments needed to improve the provision of care, a retrospective analysis has been carried out. The rate of proper referral and discharge, the number of patients followed at the hospice, the mortality rate, and the length of follow-up were analyzed, and, when possible, data were stratified by oncological and non-oncological diseases.

**Results:**

The analysis showed that of the 870 patients referred to the Padua Pediatric Hospice between 2008 and 2022, 76% were affected by non-oncological conditions. 82% of patients referred were taken in charge and most of the remaining patients have been inappropriately referred. The analysis showed a growing number of total referrals, which increased by 195% from 2008 to 2022. An increase in proper referrals and referrals of non-oncological patients was observed alongside a decrease in oncological patient referrals and a trend toward a decrease in discharge rates. A decreased mortality was observed in patients with non-oncological conditions, with only 6% of deceased patients in 2022. Moreover, a longer survival with a median follow-up length of 43 months was observed among patients with non-oncological conditions who were followed up at the Padua Pediatric Hospice for more than 12 months. Conversely, the short survival rate observed for oncological patients suggests that those patients should have been referred to PPC earlier to benefit from palliative care for longer periods.

**Conclusions:**

Considering these data, it is expected that the number of patients needing PPC services will steadily increase in the next years. Hence, there is a need to invest resources to provide the best care delivery model encompassing specific pathways for the transition into adulthood, the establishment of networks within all the Italian regions, and an efficient referral to the more suitable setting of care.

**Supplementary Information:**

The online version contains supplementary material available at 10.1186/s12904-024-01532-1.

## Background

Pediatric palliative care (PPC) globally addresses the physical and psychological needs of children with life-limiting and/or life-threatening conditions and their families, with the ultimate aim of improving their quality of life [1, 2]. Several conditions can make a child eligible for PPC, including advanced chronic non-communicable diseases, acute life-threatening conditions, severe neurodegenerative and neurological conditions, extreme prematurity, and congenital abnormalities [2, 3]. Remarkably, patients with non-oncological diseases represent most of those referring to PPC services [1–3].

According to the latest estimates, in Italy, approximately 10,600 children require specialized PPC [4]. However, the recent nationwide PalliPed project showed that less than two children out of ten have access to PPC service, suggesting that improvements in its delivery of service and better allocation of resources are eagerly required [5]. Furthermore, the Italian Law 38/2010 set off a customized response for children requiring PPC and their families to implement a network of PPC-specific services at a regional level under the coordination of a referral center. Italy is divided into 20 regions, and each one must have at least one PPC reference center and one pediatric hospice [5, 6]. According to this model, there is a specialized and interdisciplinary team in the referral center that is responsible for coordinating 24/7 the care activity of the entire network in all settings of care, including home, hospital, general PPC, or pediatric hospice, namely specialized PPC. The identification of the most adequate setting of care to which refer patients can be given by the use of appropriate tools to evaluate the PPC needs, such as the ACCAPED (Accertamento dei bisogni Clinico-Assistenziali Complessi in PEDiatria) scale [7, 8]. To achieve these goals and to allow accurate planning of services and allocation of resources, the accurate evaluation of available epidemiological data and the characterization of children in PPC are necessary [1, 5]. In particular, information on the current number of referrals and length of follow-up of patients at specialized PPC services is lacking. It also appears fundamental to differentiate between patients with oncological and non-oncological diseases since their trajectories in the PPC service are markedly different [1].

In the Italian scenario, one of the oldest centers is the PPC Referral Center of the University of Padua (Padua, Italy), which is the Reference Center of the PPC Network within the Veneto Region. The center started its activities in the first half of the 1980s and is based at the Department of Women’s and Children’s Health in Padua (University of Padua). It organizes, coordinates, and supports the activities of the PPC network of the Veneto region, which is constituted by all social and healthcare services, including hospital and local facilities, aimed at pediatric patients. The PPC Referral Center of the University of Padua coordinates integrated care responses 24/7 to the needs of children in all settings of their lives through the work of a multi-disciplinary team. While the center is the point of reference for the training and qualification of caregivers, more than 90% of the activities are carried out at home, which is the preferred place of care. In the case of special needs, the same team assists the child and his family at the Pediatric Hospice of Padua, which comprises social and health services. This pathway ensures the continuity of care, the strengthening of relationships, and the pursuit of the objectives.

Since more than 1,000 patients have been followed at the Pediatric Hospice of Padua over more than 30 years of activity, analyzing data from this specialized PPC service can provide important information on the characteristics of patients, the number of correct and inappropriate referrals, and the length of their follow-up. Here, we aim to extensively present and analyze the data of these patients over a 15-year period.

## Patients and methods

### Study design

This retrospective chart review was conducted on the patients who were referred to the Pediatric Hospice of Padua (Padua, Italy) or to the PPC network of the Veneto Region from 1 January 2008 to 31 December 2022. For the sake of brevity, from now on, the term ‘Padua Hospice’ will also include the PPC network of the Veneto region.

Eligibility criteria for specialized PPC were as follows (see Benini et al. [1] for more details): (i) being affected by a life-threatening or life-limiting disease together with complex clinical and/or psycho-social needs; (ii) risk of premature death (iii) serious episodes of hospitalization; (iv) use of invasive medical devices for life support. For patients referring to the hospice from 2020 onwards, a score > 50 on the ACCAPED scale was used as an inclusion criterion for specialized PPC [7, 8]. The Local Ethical Committee approved the study design and all Legal Guardians who could be contacted signed an informed consent to the use of their child’s data for research purposes.

### Data collection and variables considered in the analysis

Data were extracted from clinical charts and included in a dedicated spreadsheet specifically designed for this analysis and prepared with the assistance of a professional statistician. The following variables were included in the analysis: (i) the number of patients who were referred to the hospice, i.e., all patients who were referred to the hospice, whether taken in charge or not; (ii) the number of patients taken in charge by the hospice service, i.e. the proportion of patients referred to the service who were actually taken in charge; (iii) the number of patients followed at the hospice, in total and per year, i.e., patients taken in charge during the analyzed year, or during the previous years but not yet discharged; (iv) the mortality during the observation period; (v) the number of patients discharged during the observation period; and (vi) the length of follow-up per patient, i.e. the total time in which the patient was followed up by the hospice. The above variables were analyzed over the entire study period (15 years) or per year, as appropriate. When possible, results were stratified by oncological and non-oncological conditions.

### Statistical analysis

Data were analyzed by descriptive statistics using mean and standard deviation or number and percentage. The significance level was set at *p* < 0.05. The MedCalc^®^ Statistical Software version 22.016 software was used for the analysis.

## Results

### Study population

In total, 870 patients were referred to the Padua Hospice between 2008 and 2022. The mean age of children was 6.8 ± 6.3 years. Approximately three out of four patients (658/870, 76%) were affected by non-oncological conditions of which neurologic diseases were present in 152 (22%), neuromuscular disease in 130 (20%), cardiological disease in 43 (7%), lung disease in 14 (2%), adverse perinatal outcomes in 72 (11%), infectious-inflammatory disease in 22 (3%), chromosomal alterations in 35 (5%), congenital anomalies in 41 (6%) and other genetic conditions in 149 (23%) patients. The remaining 212/870 (24%) patients were affected by oncological disease, including solid tumors in 80 (38%), brain tumors in 84 (40%), and liquid tumors in 38 (18%), while definitive diagnoses were not available for 10 patients (5%). The ACCAPED median score was 62 (clinical range: 52–88) in patients with non-oncological conditions and 60 (55–93) for those with oncological diseases.

### New referrals and patients taken into charge

**Fig. 1:**
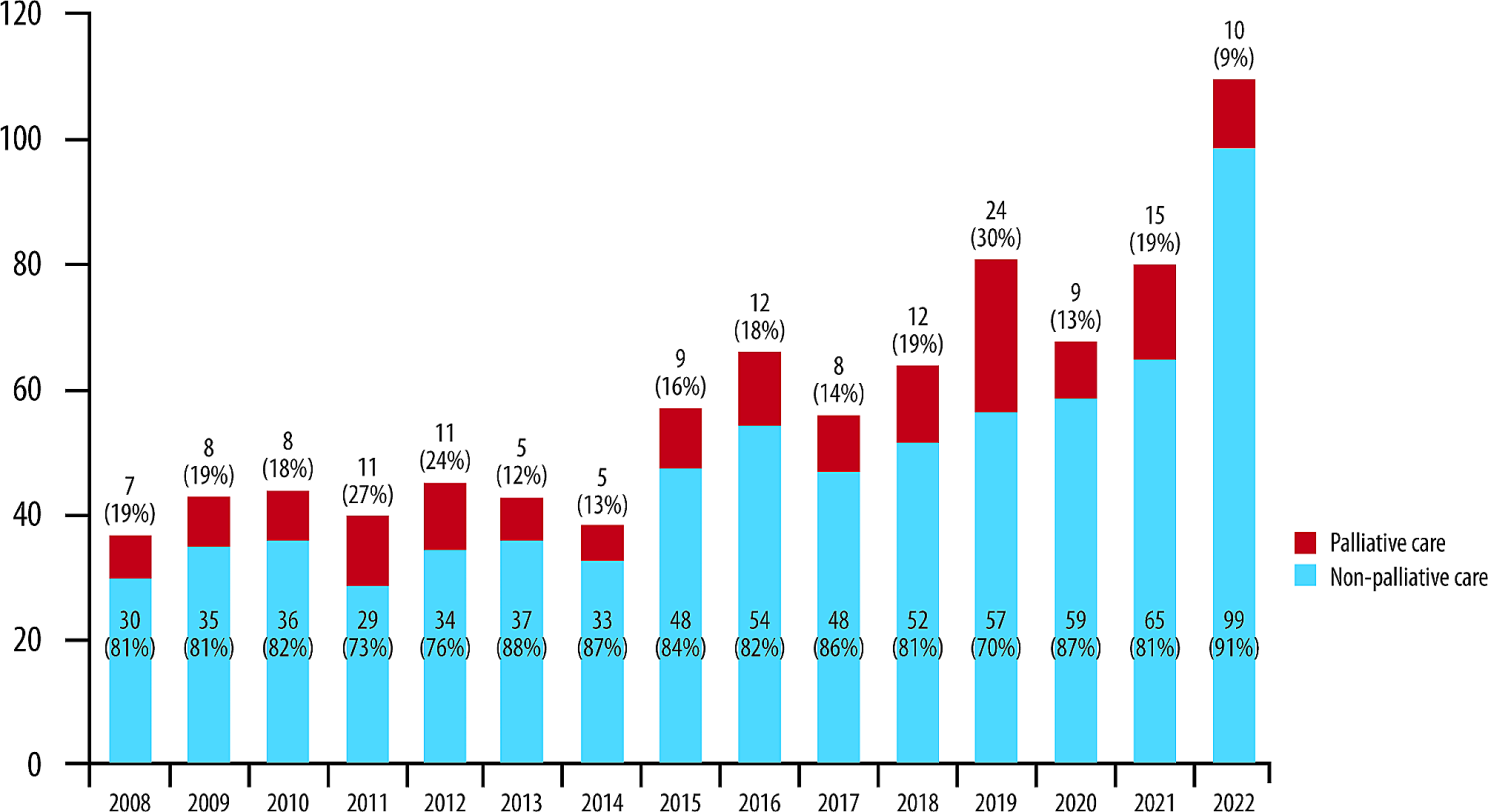
The number and percentage of new referrals and patients taken in charge over the study period by year of referral

Details on the total number of newly referred patients per year, based on the categorization in oncological or non-oncological conditions, are presented in Table 1. Overall, a steadily increasing trend was observed over time, with 109 patients referred to the hospice in 2022 compared to 37 patients referred in 2008 (195% increase). During the entire observation period, most new referrals concerned patients with non-oncological diagnoses. In total, 716/870 (82%) patients were taken in charge by the hospice service, with a trend towards an increasing proportion over time (Fig. 1). In most cases, patients were not taken in charge due to inappropriate referrals (Table 2), happening more frequently for non-oncological diagnoses than oncological. 24/870 (2.8%) patients were referred more than once (21/658, 3.2%, patients with non-oncological disease and 3/212, 1.4%, with oncological disease) due to requests for reassessments as the disease progressed or due to changes in the course of the pathology or given the increase in the needs of the patient and his family.

**Table 1:** New referrals and patients taken in charge over the observation period stratified by oncological or non-oncological diseases and by year of referral

New referrals							New patients taken in charge					
	Total	Age (years), mean (SD)	Non-oncological, *n* (%)	Non-oncological age (years), mean (SD)	Oncological, *n* (%)	Oncological age (years), mean (SD)	Total	Age (years), mean (SD)	Non-oncological, *n* (%)	Non-oncological age (years), mean (SD)	Oncological, *n* (%)	Oncological age (years), mean (SD)
2008	37	6.0 (± 5.9)	25 (68)	5.2 (± 5.8)	12 (32)	7.8 (± 6.1)	30	5.1 (± 5.4)	19 (63)	4.1 (± 5.4)	11 (37)	6.8 (± 5.3)
2009	43	6.1 (± 5.8)	34 (79)	5.1 (± 5.6)	9 (21)	9.9 (± 5.2)	35	5.8 (± 6.0)	28 (80)	4.8 (± 5.8)	7 (20)	10.0 (± 5.2)
2010	44	5.4 (± 5.0)	27 (61)	3.3 (± 3.9)	17 (39)	8.6 (± 5.0)	36	5.7 (± 5.2)	22 (61)	3.7 (± 4.0)	14 (39)	8.9 (± 5.4)
2011	40	5.9 (± 5.5)	32 (80)	5.2 (± 5.4)	8 (20)	8.6 (± 5.5)	29	6.0 (± 5.4)	22 (76)	4.8 (± 5.0)	7 (24)	9.6 (± 5.1)
2012	45	6.8 (± 7.0)	32 (71)	5.3 (± 6.0)	13 (29)	10.4 (± 8.4)	34	6.0 (± 5.4)	23 (68)	5.3 (± 5.5)	11 (32)	7.6 (± 5.1)
2013	42	6.8 (± 6.8)	33 (79)	5.9 (± 6.7)	9 (21)	10.3 (± 6.2)	37	6.2 (± 6.7)	29 (78)	5.2 (± 6.4)	8 (22)	10.1 (± 6.6)
2014	38	4.0 (± 5.3)	31 (82)	3.3 (± 5.2)	7 (18)	7.4 (± 4.8)	33	3.4 (± 5.0)	28 (85)	2.8 (± 4.9)	5 (15)	7.8 (± 3.9)
2015	57	6.0 (± 5.6)	45 (79)	5.0 (± 5.5)	12 (21)	9.9 (± 4.6)	48	5.7 (± 5.4)	38 (79)	4.6 (± 5.1)	10 (21)	10.1 (± 4.4)
2016	66	5.9 (± 6.1)	49 (74)	5.3 (± 6.5)	17 (26)	7.7 (± 4.2)	54	5.6 (± 5.7)	39 (72)	4.7 (± 6.1)	15 (28)	7.8 (± 3.9)
2017	56	8.6 (± 6.6)	34 (61)	5.7 (± 6.1)	22 (39)	13.1 (± 4.6)	48	8.9 (± 6.7)	29 (60)	6.0 (± 6.3)	19 (40)	13.4 (± 4.4)
2018	64	7.7 (± 6.6)	45 (70)	6.8 (± 6.9)	19 (30)	10.0 (± 5.6)	52	6.7 (± 5.6)	36 (69)	5.6 (± 5.8)	16 (31)	9.1 (± 4.7)
2019	81	6.6 (± 6.3)	67 (83)	5.8 (± 6.2)	14 (17)	10.4 (± 5.9)	57	6.5 (± 6.2)	47 (83)	5.5 (± 5.9)	10 (18)	11.3 (± 5.4)
2020	68	6.2 (± 5.8)	47 (69)	5.5 (± 5.9)	21 (31)	7.8 (± 5.3)	59	6.3 (± 5.8)	38 (64)	5.4 (± 5.9)	21 (36)	7.8 (± 5.3)
2021	80	8.0 (± 6.6)	65 (81)	7.0 (± 6.1)	16 (19)	12.2 (± 7.1)	65	8.3 (± 6.6)	51 (79)	6.8 (± 5.9)	14 (22)	13.8 (± 6.1)
2022	109	7.8 (± 6.7)	93 (85)	7.1 (± 6.7)	16 (15)	11.4 (± 5.7)	99	7.7 (± 6.7)	84 (85)	7.1 (± 6.7)	15 (15)	11.4 (± 5.9)
**Total**	**870**	**6.8 (± 6.3)**	**658 (76)**	**5.8 (± 6.1)**	**212 (24)**	**9.9 (± 5.8)**	**716**	**6.5 (± 6.1)**	**533 (74)**	**5.4 (± 5.9)**	**183 (26)**	**9.8 (± 5.4)**

**Table 2:** Patients not taken in charge over the study period

Year	Not taken in charge			Improper referral			Others		
	Total, *n* (%)	Non-oncological, *n* (%)	Oncological, *n* (%)	Total, *n* (%)	Non-oncological, *n* (%)	Oncological, *n* (%)	Total, *n* (%)	Non-oncological, *n* (%)	Oncological, *n* (%)
2008 (*n* = 37)	7 (19)	6 (16)	1 (3)	7 (19)	6 (16)	1 (3)	–	–	–
2009 (*n* = 43)	8 (19)	6 (14)	2 (5)	8 (19)	6 (14)	2 (5)	–	–	–
2010 (*n* = 44)	8 (18)	5 (11)	3 (7)	7 (16)	5 (12)	2 (5)	1 (2)	–	1 (2)
2011 (*n* = 40)	11 (28)	10 (25)	1 (3)	9 (23)	8 (20)	1 (2)	2 (5)	2 (5)	–
2012 (*n* = 45)	11 (24)	9 (20)	2 (4)	10 (22)	8 (18)	2 (5)	1 (2)	1 (2)	–
2013 (*n* = 42)	5 (12)	4 (10)	1 (2)	4 (10)	3 (7)	1 (2)	1 (2)	1 (2)	–
2014 (*n* = 38)	5 (13)	3 (8)	2 (5)	5 (13)	3 (8)	2 (5)	–	–	–
2015 (*n* = 57)	9 (16)	7 (12)	2 (4)	8 (14)	6 (11)	2 (4)	1 (2)	1 (2)	–
2016 (*n* = 66)	12 (18)	10 (15)	2 (3)	9 (14)	7 (11)	2 (3)	3 (5)	3 (5)	–
2017 (*n* = 56)	8 (14)	5 (9)	3 (5)	6 (11)	4 (7)	2 (3)	2 (3)	1 (2)	1 (2)
2018 (*n* = 64)	12 (19)	9 (14)	3 (5)	8 (13)	6 (9)	2 (3)	4 (6)	3 (4)	1 (2)
2019 (*n* = 81)	24 (30)	20 (25)	4 (5)	19 (23)	16 (20)	3 (4)	5 (6)	4 (5)	1 (1)
2020 (*n* = 68)	9 (13)	9 (13)	–	8 (12)	8 (12)	–	1 (2)	1 (2)	–
2021 (*n* = 80)	15 (19)	13 (16)	2 (3)	12 (15)	10 (13)	2 (3)	3 (4)	3 (4)	–
2022 (*n* = 109)	10 (9)	9 (8)	1 (1)	10 (9)	9 (8)	1 (2)	–	–	–

### Patients followed at the hospice

**Fig. 2:**
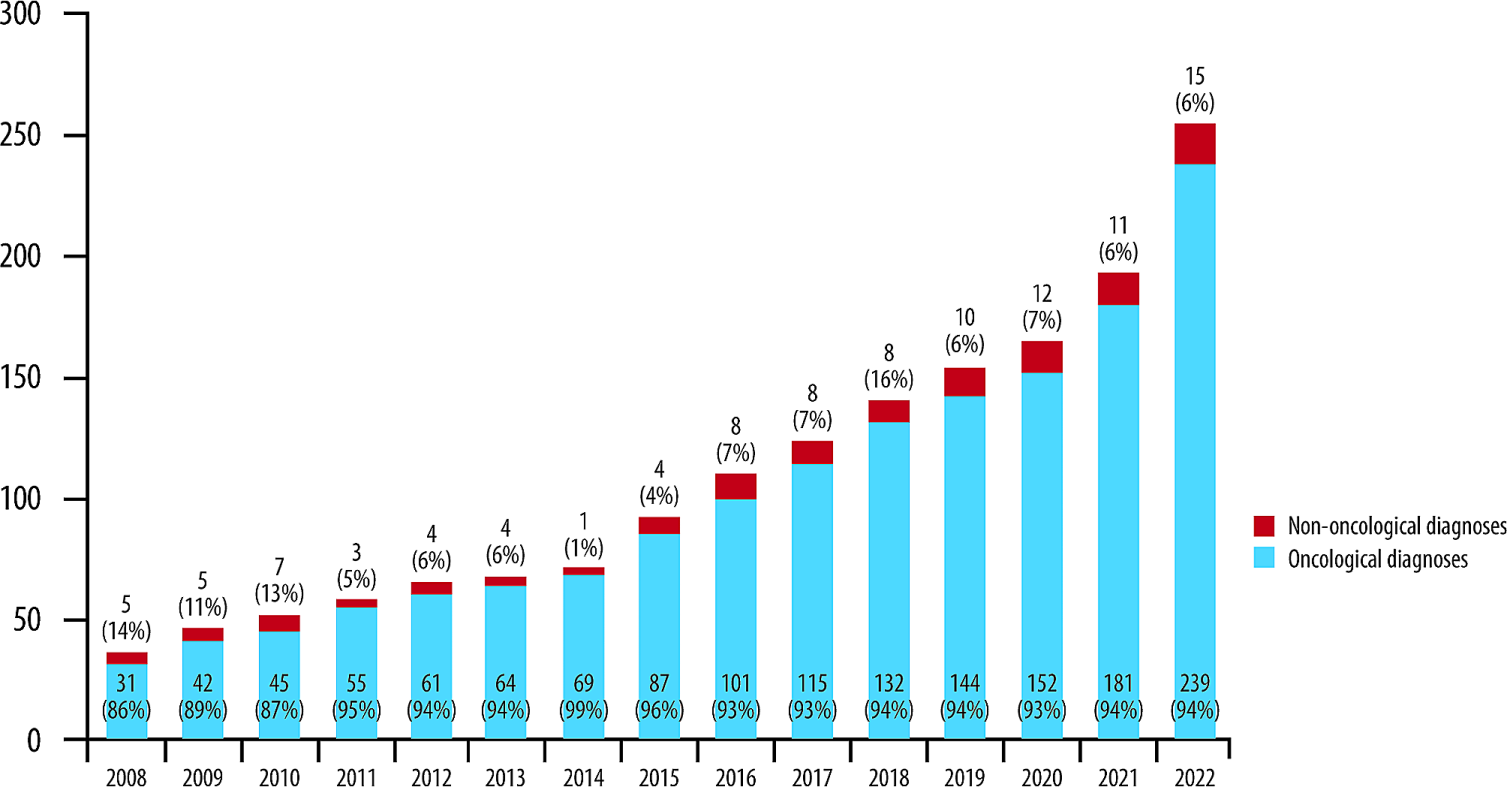
Patients followed at the hospice over the observation period stratified by oncological and non-oncological diseases and by year

The total number of patients followed each year at the hospice increased each year, from 58 in 2008 to 291 in 2022, while the proportion of patients with oncological disease decreased (Fig. 2).

### Mortality during the observation period

Over the observation period, 383 out of the 716 patients taken in charge died, resulting in an overall mortality rate of 53%. As presumable, this rate was higher in oncological conditions (165/183, 90%) compared to non-oncological disorders (218/533, 41%). Interestingly, a decreasing trend in the mortality rate was observed over time (Table 3).

**Table 3:** Mortality rate over the observation period

Year	Total, *n* (%)	Non-oncological, *n* (%)	Oncological, *n* (%)
2008	15/58 (26)	3/41 (7)	12/17 (71)
2009	14/70 (20)	7/58 (12)	7/12 (58)
2010	21/81 (26)	10/62 (16)	11/19 (58)
2011	18/80 (23)	8/66 (12)	10/14 (71)
2012	21/92 (23)	13/78 (17)	8/14 (57)
2013	26/102 (25)	19/90 (21)	7/12 (58)
2014	22/100 (22)	15/92 (16)	7/8 (88)
2015	23/118 (19)	16/107 (15)	7/11 (64)
2016	32/145 (22)	21/126 (17)	11/19 (58)
2017	29/157 (18)	11/130 (8)	18/27 (67)
2018	30/175 (17)	15/151 (10)	15/24 (63)
2019	34/197 (17)	26/179 (15)	8/18 (44)
2020	40/212 (19)	22/181 (12)	18/31 (58)
2021	32/229 (14)	17/203 (8)	15/26 (58)
2022	26/291 (9)	15/265 (6)	11/26 (42)
**Total**	**383/716 (53)**	**218/533 (41)**	**165/183 (90)**

The mean age at death was 7.1 ± 6.0 years, considering that patients affected by non-oncological conditions died at a younger age (Table S1).

### Number of patients discharged during the observation period

Over the observation period, 108/716 patients were discharged since they showed lower complexity of needs (15%), with a mean age of 9.1 ± 7.4 years (Table S2). This occurred in 18% of the non-oncological cases (98/533) and 5% of the oncological ones (9/183). A decreasing trend in the discharge rate per year was observed over time (Table 4).

**Table 4:** Discharge rate over the observation period

Year	Total, *n* (%)	Non-oncological, *n* (%)	Oncological, *n* (%)
2008	8/58 (14)	8/41 (20)	0/17 (0)
2009	9/70 (13)	9/58 (16)	0/12 (0)
2010	10/81 (12)	9/62 (15)	1/19 (5)
2011	4/80 (5)	3/66 (5)	1/14 (7)
2012	6/92 (7)	4/78 (5)	2/14 (14)
2013	8/102 (8)	7/90 (8)	1/12 (8)
2014	8/100 (8)	8/92 (9)	0/8 (0)
2015	4/118 (3)	4/107 (4)	0/11 (0)
2016	4/145 (3)	4/126 (3)	0/19 (0)
2017	5/157 (3)	4/130 (3)	1/27 (4)
2018	5/175 (3)	4/151 (3)	1/24 (4)
2019	9/197 (5)	9/179 (5)	0/18 (0)
2020	10/212 (5)	9/181 (5)	1/31 (3)
2021	6/229 (3)	5/203 (2)	1/26 (4)
2022	11/291 (4)	11/265 (4)	0/26 (0)
**Total**	**108/716 (15)**	**98/533 (18)**	**9/183 (5)**

### Length of follow-up

Table 5 depicts the distribution of follow-up length over the observation period. In total, 39% of patients (277/716) were followed for > 12 months. This percentage rose to 52% for non-oncological diseases (275/533). Of the remaining 48% of children with non-oncological disease (258/533) who had a follow-up < 12 months, a good proportion (93, 34%) were referred to the hospice in 2022, and, therefore, their follow-up was not concluded. Considering the non-oncological population followed for > 12 months, the median duration of the follow-up was 43 months (95% CI: 37–47 months). Within the oncological population, 91% of children had a follow-up < 12 months (167/183) with a median of 1.8 months (95% CI: 1.4–2.7 months).

**Table 5:** Distribution of the length of follow-up

Duration (months)	Total, *n* (%)	Non-oncological, *n* (%)	Oncological, *n* (%)
< 12	439 (61.0)	275 (52.0)	164 (90.0)
12–24	80 (11.0)	69 (13.0)	11 (6.0)
24–36	48 (7.0)	44 (8.0)	4 (2.0)
36–48	37 (5.0)	36 (7.0)	1 (0.5)
48–60	26 (4.0)	25 (5.0)	1 (0.5)
60–72	13 (2.0)	13 (2.0)	-
72–84	21 (3.0)	19 (4.0)	2 (1.0)
84–96	11 (2.0)	11 (2.0)	-
96–108	8 (1.0)	8 (2.0)	-
108–120	3 (0.4)	3 (0.6)	-
≥ 120	30 (4.0)	30 (6.0)	-
**Total**	**716**	**533**	**183**

In a 5-year timeframe, the proportion of patients with ongoing follow-up increased over time (Table 6). Remarkably, among the patients still receiving care in 2022, 13% (37/291) and 28% (81/291) were taken in charge more than 10 or more than 5 years earlier, respectively.

**Table 6:** Patients still followed-up over 5-year windows

5-year period	Total	Patients still receiving care at the end of 5 years, *n* (%)
2012–2008	164	65 (40)
2013–2009	171	68 (40)
2014–2010	169	70 (41)
2015–2011	181	91 (50)
2016–2012	206	109 (53)
2017–2013	220	123 (56)
2018–2014	235	140 (59)
2019–2015	259	154 (59)
2020–2016	270	164 (61)
2021–2017	281	192 (68)
2022–2018	332	254 (77)

## Discussion

Given the overall poor coverage on the Italian territory, the PPC service in Italy needs to be further implemented through the establishment of PPC networks [4, 5]. Hence, a proper allocation of resources on the base of an accurate estimation of the actual burden of needs and the characterization of children requiring PPC in Italy is needed [1, 5].

Intending to gather information on the number of referrals and the follow-up length of patients in PPC, we retrospectively analyzed the information of the patients who were referred to the Padua Hospice over a 15-year period.

Among the 870 patients who were referred to the Padua Hospice between 2008 and 2022, the majority (76%) were affected by non-oncological conditions. This finding is not unexpected since it is in line with previous reports in the PPC setting [1, 5, 9, 10] and contradicts, at least in part, the common notion that PPC focuses on the ‘care of children with oncological disease at a terminal stage’ [1].

Notably, PPC patients affected by non-oncological conditions showed better survival rates than those with oncological disease (41% vs. 90%) since non-oncological conditions do not cause deaths as much as cancer does. For this reason, their follow-up in PPC was much longer (43 months vs. 1.8 months). Survival rates also increased over time. Indeed, approximately 40% of patients taken in charge are followed for more than 1 year and a remarkable proportion of children (28%) for more than five. Such a long period of follow-up translates into a continuous change in the needs of the patients due to the unpredictable course of some pathologies, the different phases of the condition or disease progression and due to the transition into adulthood [1].

Another expected finding is the short follow-up for patients in PPC with oncological conditions, whose mortality rate was also very high (90%). Since the overall survival rate for children with oncological disease not in PPC is increasing worldwide [11, 12], it is possible to speculate that, in our setting of care, children with cancer are referred to PPC only at a very late stage of the disease. It is possible to suggest that earlier referral would translate into a better quality of life for the patient and his/her family without any adverse impact on survival [11–13].

The analysis of the referrals per year shows a steady increase over the study period: in 2022, more than 100 patients were referred to our network, a 195% increase compared to 2008. The same increasing trend could be observed, at least in part, for the number of patients actually taken in charge (91% in 2022). These findings disclose an improvement in the overall implementation of the PPC in the Veneto Region, with higher coverage and more accurate referral of patients to be followed by a specialized service. Indeed, it is worth noting that in recent years, in the Veneto region, training has been given to PPC providers to support the identification of eligible patients and the activation of the PPC network and to educate them for integrated working. This certainly had a good impact on the activity of the network and the quality of the assistance provided. Also, the increased number of patients taken in charge of the Padua Hospice and their increasing survival account for the increasing number of patients followed each year (291 in 2022). It has to be considered that the longer survival of patients observed implies the necessity for the healthcare system to monitor the patients’ needs and dynamically and effectively adapt its response. This is the pathway followed until an eventual discharge if the patient does not require specialist intervention anymore, which in our data occurred in 10% of cases per year. These data highlight the importance of ensuring the enrollment and discharge of patients from specialized PPC by a shared and validated assessment (ACCAPED) of the individual patient’s needs [7].

Furthermore, there is an increase in the number of long-surviving pediatric patients who reach adulthood. Indeed, 16% of patients in charge of the Italian network are over 16 years old [14]. This emerging problem triggers the need for the establishment of an appropriate care model for the transition to adult services after having appropriately evaluated data, needs, resources, and available healthcare models. Currently, there is no determination of the age at which start the transition to adult palliative care [15]. In the UK patients are referred to specialist pediatric care from age 16 to 19 years before transitioning [16], and the transition is planned to occur around 18 years in the USA [17]. However, older patients with very complex syndromes can still be referred to pediatric palliative care services [18]. Collectively, these findings highlight a major burden for the PPC network in the Veneto Region, also increasing over time (a preliminary analysis of patients followed in 2023, still immature, seems to confirm the overall results). Data collected in a nationwide cross-sectional study also confirm the high number of patients followed by PPC services in Italy [14].

This prompts an adequate allocation of resources and, at the same time, the establishment of dedicated educational curricula for healthcare providers potentially involved in PPC [1, 14]. Moreover, educative and awareness campaigns should be implemented since parents/patients are still afraid of accessing services because they believe that palliative care is treatment aimed only at dying children. In addition, appropriate communication focused on the role of PPCs in providing treatments to manage incurable diseases and on the rights that each child and his/her family have in this area could help address the cultural challenge around PPC services.

Although our data were collected retrospectively, the present study can represent the basis for the prospective collection of data that will help keep track of the burden of PPC and monitor the epidemiology of patients. Indeed, further studies are needed to collect data on patients who have been recently taken into charge by PPC services.

It could also be helpful to extend this study design to national and international levels to collect comprehensive data on this often-neglected research field and to consolidate PPC networks.

### Electronic supplementary material

Below is the link to the electronic supplementary material.

Supplementary Material 1

## Data Availability

The data that support the findings of this study are available on request from the corresponding author.

## Abbreviations

ACCAPED: Accertamento dei bisogni Clinico-Assistenziali Complessi in PEDiatria
PPC: Pediatric palliative care
