# Supplementary material for: A 15-year experience in pediatric palliative care: a retrospective hospital-based study
Source: BMC Palliat Care. 2024 Aug 6;23:202. doi: 10.1186/s12904-024-01532-1 (PMC11301942; doi:10.1186/s12904-024-01532-1)
Supplement: Supplementary file 1 — Supplementary Material 1 [file 12904_2024_1532_MOESM1_ESM.docx]

**Table S1. Mean age at death.**

| **Year** | **Total, mean (SD)** | **Non-oncological, mean (SD)** | **Oncological, mean (SD)** |
| --- | --- | --- | --- |
| 2008 | 6.0 (±5.9) | 0.5 (±0.02) | 7.4 (±5.9) |
| 2009 | 7.0 (±6.5) | 3.7 (±5.9) | 10.2 (±5.7) |
| 2010 | 6.9 (±5.4) | 3.7 (±4.3) | 9.9 (±4.6) |
| 2011 | 5.7 (±5.6) | 3.3 (±5.9) | 7.6 (±4.9) |
| 2012 | 6.6 (±5.8) | 3.9 (±3.8) | 11.0 (±6.0) |
| 2013 | 5.1 (±5.1) | 4.2 (±5.0) | 7.7 (±5.0) |
| 2014 | 4.2 (±4.9) | 2.7 (±4.1) | 7.5 (±5.0) |
| 2015 | 5.3 (±5.6) | 3.3 (±4.7) | 9.8 (±5.1) |
| 2016 | 7.5 (±6.3) | 7.0 (±7.2) | 8.4 (±4.0) |
| 2017 | 9.8 (±5.0) | 5.8 (±4.5) | 12.3 (±3.7) |
| 2018 | 9.3 (±6.7) | 7.2 (±7.0) | 11.5 (±5.8) |
| 2019 | 6.1 (±5.6) | 4.4 (±4.8) | 11.5 (±4.5) |
| 2020 | 7.7 (±5.9) | 6.6 (±5.8) | 9.1 (±5.9) |
| 2021 | 7.5 (±6.3) | 4.1 (±5.6) | 11.4 (±4.5) |
| 2022 | 8.7 (±7.2) | 5.8 (±7.5) | 12.8 (±4.3) |

**Table S2. Mean age at discharge over the observation period.**

| **Year** | **Total, mean (SD)** | **Non-oncological, mean (SD)** | **Oncological, mean (SD)** |
| --- | --- | --- | --- |
| 2008 | 8.8 (±6.2) | 8.8 (±6.2) | - |
| 2009 | 8.3 (±6.8) | 8.3 (±6.8) | - |
| 2010 | 8.1 (±5.5) | 8.0 (±5.9) | 9.1 |
| 2011 | 8.3 (±6.8) | 6.2 (±6.7) | 14.4 |
| 2012 | 10.2 (±8.3) | 9.9 (±8.3) | 10.8 (±11.5) |
| 2013 | 14.3 (±4.4) | 14.5 (±4.8) | 13.6 |
| 2014 | 6.2 (±7.1) | 6.2 (±7.1) | - |
| 2015 | 5.1 (±5.4) | 5.1 (±5.4) | - |
| 2016 | 9.2 (±7.8) | 9.2 (±7.8) | - |
| 2017 | 5.1 (±5.4) | 5.0 (±6.3) | 5.4 |
| 2018 | 6.3 (±5.7) | 3.9 (±1.8) | 16.1 |
| 2019 | 10.4 (±8.0) | 10.4 (±8.0) | - |
| 2020 | 8.1 (±7.3) | 6.9 (±6.6) | 19.0 |
| 2021 | 9.4 (±10.7) | 5.7 (±6.2) | 28.2 |
| 2022 | 12.9 (±10.0) | 12.9 (±10.0) | - |
